# Effect of calorie-restriction and rapamycin on autophagy and the severity of caerulein-induced experimental acute pancreatitis in mice

**DOI:** 10.3389/fgstr.2022.977169

**Published:** 2022-10-12

**Authors:** Manish Kumar Sharma, Kumari Priyam, Punit Kumar, Pramod Kumar Garg, Tara Sankar Roy, Tony George Jacob

**Affiliations:** ^1^ Department of Anatomy, All India Institute of Medical Sciences, New Delhi, India; ^2^ Department of Gastroenterology, Translational Health Science and Technology Institute, Faridabad, India; ^3^ Department of Anatomy, North Delhi Municipal Corporation (DMC) Medical College & Hindu Rao Hospital, New Delhi, India

**Keywords:** autophagic flux, apoptosis, inflammation, necrosis, acute pancreatitis

## Abstract

**Background:**

Impaired autophagy contributes to development of acute pancreatitis (AP). We studied the effect of inducing autophagy by calorie-restriction and rapamycin, separately, in the caerulein-induced model of severe AP.

**Methods:**

Adult, male, Swiss albino mice were given eight, hourly, intraperitoneal injections of caerulein (Ce) (50µg/Kg/dose). The interventions were calorie restriction (CR) and rapamycin (2mg/Kg). Mice were sacrificed at the 9^th^ hour. Pancreas was harvested for histopathology and immunoblotting. Amylase activity and the levels of cytokines were measured in plasma.

**Results:**

The histopathological score and amylase activity were significantly lower in calorie-restricted caerulein-induced AP (CRCeAP) in comparison to animals that had unrestricted access to chow. In the CRCeAP group, levels of IL-6 and GM-CSF in plasma were lower and the expression of LC3II and Beclin-1 were higher. On transmission electron-microscopy, the area occupied by autophagic vacuoles was higher in CRCeAP. The expression of caspase-8 and caspase-9 was also higher in CRCeAP. In rapamycin with caerulein-induced AP (Rapa+CeAP), the histopathological score and amylase activity were significantly lower than caerulein-induced AP (CeAP). In Rapa+CeAP, the expression of LC3II and Beclin-1 were higher, whereas; SQSTM1 was decreased. The number of autophagic vacuoles in Rapa+CeAP group was fewer. Interleukin-6 (IL-6), tumor necrosis factor-α (TNF-α) and monocyte chemoattractant protein-1 (MCP-1) were lower in Rapa+CeAP. Caspase-3 increased and high mobility group box 1 (HMGB1) decreased in Rapa+CeAP.

**Conclusion:**

Calorie-restriction and rapamycin can individually decrease the severity of injury in the caerulein-induced model of severe AP.

## Introduction

Acute Pancreatitis (AP) is an acute inflammatory disease of the exocrine pancreas that has an incidence of 13-45 per 100,000 persons (1). The etiology of AP is varied (2) but the exact pathogenesis of AP is still not well understood (3). It is known that acinar cell stress may lead to cellular injury and death. The necrotic form of cell death leads to more inflammation and the extent of necrosis is a histopathological marker of severity of AP (4).

The role of cellular stresses particularly oxidative and endoplasmic reticulum (ER) stress has been recently highlighted in the pathogenesis of AP (5). ER stress as a result of unfolded/misfolded proteins has been shown in different experimental models of AP and also in human pancreatitis (6, 7). The responses to cellular stresses are multiple, but they all attempt to re-establish homeostasis. One of the fundamental homeostatic mechanisms associated with AP is autophagy. Autophagy allows the cell to remove damaged protein and organelles through a regulated lysosomal-dependent mechanism. Several proteins are required for the autophagic process such as LC3 (LC3 gets lipidylated at the onset of autophagy and converted to LC3II). Beclin1, is required for the induction of autophagy, and SQSTM1, is a ubiquitin binding protein that is expressed on the inner membrane of phagophore. After completion of the autophagic flux, the amount of SQSTM1 reduces because it is also degraded along with the rest of the cargo within the autophagosome (8). Different physiological or pathological stimuli such as starvation, depletion of amino acids, decreased growth factors, hypoxia, DNA damage, ER stress and infection can induce autophagy (9–11).

Gukovskaya et al. (2012) (12) showed that there was evidence of impaired autophagy in experimental AP, which leads to accumulation of autophagic vesicles and activation of death pathways. Since, calorie-restriction for 18-24 hours is the norm during the induction of experimental pancreatitis in various models of AP (4, 13–15); this may induce autophagy in most cells, including the pancreatic acinar cells. Thus, it may be a confounding variable in experimental AP. During calorie-restriction, there is activation of AMP-activated protein kinase (AMPK), which acts as an energy sensor in cells and plays a key role in the activation of the autophagic process. Under nutrient deficient conditions, AMPK is phosphorylated by an upstream kinase and then it binds to AMP rather than ATP. This activates mTORC1, which has been reported as a key in the modulation of autophagy signaling. In presence of sufficient nutrients there is activation of mTORC1 and this keeps autophagy inhibited (16). Rapamycin is a drug that inhibits the activation of mTORC1 and hence, can induce autophagy (17).

There is some controversy about the role of autophagy because some groups have suggested that impaired autophagy contributes to worsening of pancreatitis (7, 18) while others have shown that inhibition of autophagy is beneficial (19). It is known that rapamycin and calorie-restriction can induce autophagy (16, 20, 21) but there is little evidence about the therapeutic effect of modulating autophagy on disease progression in severe AP. Hence, in the present study, we evaluated the effect of modulation of autophagy on the severity of AP in the caerulein-induced experimental model of AP.

## Materials and methods

We conducted the experiments after obtaining ethical clearance from the Institutional Animal Ethics Committee (Ref. No. 941/IAEC/16). We used adult, male, Swiss albino mice weighing 20-25 grams (4 to 6 weeks old) that were kept in shoe-box cages in a 12-hour day-night cycle and provided food and water *ad libitum* till the day of the experiment. The animals were randomly allocated to one of four groups that had six mice each.

Severe AP was induced in the mice by eight, hourly intraperitoneal injections of caerulein (Cat No. C9026, Sigma, USA) that was dissolved in normal saline, and given at a dose of 50µg/Kg body weight. The control animals received equivalent volumes of normal saline. The mice were sacrificed in a CO_2_ chamber, one-hour after the 8^th^ injection. At the time of sacrifice, blood was withdrawn from the right ventricle into a heparinized syringe. Plasma was separated by centrifuging the heparinized blood at 3000 rpm for 10 min at 4°C. The supernatant was pipetted out, aliquoted and stored at -80°C for further analysis. The pancreas was carefully dissected out and a part of it was fixed by immersion in chilled buffered paraformaldehyde {made in 0.1M phosphate buffer (PB), pH 7.4} for histopathology, and in modified Karnovsky’s solution (2.5% glutaraldehyde, 2% paraformaldehyde in 0.1M PB, pH 7.4) for transmission electron microscopy (TEM). The rest was flash frozen in liquid nitrogen and stored at -80°C till further analysis.

### Caerulein-induced severe AP after physiological induction of autophagy by calorie restriction (CR)

Severe AP was induced by caerulein as described above, either after calorie restriction or after free access to chow during the night before induction of AP. In addition, there were two groups of control mice: one with calorie restriction (CR control) and other with overnight free access to chow but both were given saline instead of caerulein. Overall mice were randomly divide into four groups (n=6 each) - Calorie restricted control (CR Control), night feeding control (NFe Control), Calorie restricted caerulein-induced AP(CRCeAP) and night feeding with caerulein-indcued AP (NFeCeAP) (**Supplementary Figure 1**).

We measured the level of glucose in the blood taken from the tail vein of the mice using ACCU-CHEK kit (Roche Diabetes Care, Mumbai, India) at three different time points during the experiment. First, the baseline of blood glucose level was estimated at 5.30 PM, i.e.14-hours prior to the induction of pancreatitis in the experimental mice, then at 7.00 AM (30-minutes before the first injection) and at the time of sacrifice of the mice at 5.30 PM.

### Caerulein-induced severe AP after pharmacological induction of autophagy by rapamycin

Mice were divided in four groups – control, rapamycin control, caerulein-induced AP (CeAP), and rapamycin with caerulein-induced AP (Rapa+CeAP). The control group was given intraperitoneal injections of normal saline. The rapamycin group was given intraperitoneal injections of rapamycin at 14-hours and 30-minutes prior to injections of either normal saline or caerulein. The dose of rapamycin was 2 mg/Kg (Cat No. R0395, Sigma, USA), which was dissolved in dimethyl sulfoxide (DMSO) as has been described before by Wan et al. (2018) (22) (**Supplementary Figure 2**).

### Assessment of pancreatitis and autophagy

The fixed pancreas was washed, dehydrated in ascending grades of alcohol, cleared in chloroform and embedded in paraffin. We prepared paraffin blocks and these were sectioned at 5 µm thicknesses, floated on a water-bath (maintained at 40°C) and taken on clean glass-slides, coated with egg albumin. At least six (non-serial) sections were taken per tissue per animal. The sections were stained with hematoxylin and eosin and mounted with dibutylphthalate polystyrene xylene (DPX) (4). The sections were scored under the 40X-objective of a light microscope (Olympus BX61, Japan), attached to an imaging software through which images were also obtained. The scoring of pancreatic injury was performed according the parameters described by Schmidt et al. (1992) (23) (maximum score of 16) by two trained pathologists, who were blinded to the identity of the tissue sections.

For TEM, 1-mm^3^ tissue blocks were fixed in modified Karnovsky’s solution, post-fixed in 1% OsO_4_ (in 0.1M PB, pH 7.4), dehydrated in ascending grades of acetone and embedded and blocked in araldite CY212. Semithin sections (0.5 µm thick sections) were cut initially and stained with toluidine blue (1% aqueous solution) to determine the area of interest and guide the trimming of the resin block. Ultrathin sections (silver) were cut on an ultramicrotome (Reichert-Jung, Leica, Massachussetts, USA) and collected on 300-mesh copper grids, stained with uranyl acetate and lead citrate and viewed under Tecnai G^2^- 20 TEM (FEI, Netherlands). Images were captured using digital micrograph software (USA) (24).

The TEM images were used for quantifying autophagic vacuole count and autophagic area occupied by vacuoles to identify the completion or impaired autophagy after calorie-restriction and pre-treatment with rapamycin. The mean magnification was 2550X. All vacuoles were manually counted and their area was measured from at least 24 different fields for each animal in each group (n=3/group). At first, the ImageJ software (1.52a, NIH, USA) was calibrated using the embedded scale bar (1µm). The total area of the image, area of vacuole (single membrane structure), area of nucleus and area of interstitial space were measured using separate contour lines that were drawn around each of these entities. After that we deducted the area of nucleus and area of interstitial space from the total area to obtain the area occupied by the cytoplasm. The total area of all vacuoles was divided by the area of cytoplasm and multiplied by hundred to calculate the percentage area of cytoplasm occupied by vacuoles.

For immunoblotting, initially the samples of pancreas stored at -80°C were weighed and homogenized in 1.5 mL of RIPA buffer (Cat. No. 89900, Thermo Scientific, USA) with a protease inhibitor cocktail (1% volume/volume) (Cat. No. 88265, Thermo Scientific, USA). The homogenates were kept at 4°C for an hour, after which they were centrifuged at 20,000g at 4°C for 30-minutes. The supernatant was pipetted out and it was further centrifuged for 30-minutes at 20,000g at 4°C. The last supernatant was aliquoted and stored at -80°C and a small aliquot was also used to quantify the protein content in it by the Bradford’s method using a multiplate reader (BioTek, USA) at 595 nm. Thereafter, 30 µg of the protein was denatured with β-mercaptoethanol and loaded into a well in a 10% polyacrylamide gel and separated electrophoretically at a constant current of 15 mAmp. The separated proteins in the gel were transferred to a polyvinylidene fluoride (PVDF) membrane (0.22 µm pore size, Milipore, USA) at a constant voltage of 70mV. The membranes were then first blocked in 3% bovine serum albumin and later probed with primary and secondary antibodies and chemiluminescent reagent for the presence of the proteins of our interest. We used β-actin (Conc-1:5000, Abcam, Cat. No. ab6276, UK) as a loading control to calculate the relative expression of the proteins. The membranes were developed and photographed through enhanced chemiluminescence method in Fluor Chem-M (Protein simple, Newark, CA). The intensity of the protein bands was quantified by ImageJ software (1.52a, NIH, USA).

The induction of autophagy was estimated by the expression of LC3II (Conc-1:800, Abcam, Cat. No. ab128025, UK) and Beclin1 (Conc- 1:1000, Abcam, Cat No. ab207612, UK) (25); and completion of the autophagic flux by the expression of Sequestosome 1(SQSTM1) (Conc- 1:1000, Abcam, Cat. No. ab56416, UK) (26). The inherent inflammatory response was estimated by the expression of Receptor-interacting serine/threonine-protein kinase 1 (RIPK1), which is an integral part of the inflammasome complex (27–29) (Conc- 1:1000, Abcam, Cat No. ab72139, UK). The expression of NF-κB is estimated by the expression of its component P65 (Conc- 1:1000, Cell Signaling Technology, Cat No. 8242, USA). The extent of apoptosis was estimated by the relative expression of caspase-3 (Conc-1:1000, Abcam, Cat. No. ab13847, UK), caspase-8 (Conc- 1:1000, Abcam, Cat No. ab119892, UK) and caspase-9 (Conc- 1:2000, Cell Signaling Technology, Cat No. 9508, USA). For necrosis, we used the relative expression of High mobility group box 1 (HMGB1) protein (Conc-1:1000, Abcam, Cat. No. ab184203, UK), which is a nuclear protein. Increased level of HMGB1 in the extracellular space is considered to be an indicator of necrotic cell death (30).

Amylase activity was measured in plasma by an enzymatic colorimetric test (Centronic GmbH, Cat No. AF03000060) according to the manufacturer’s instructions. Optical density was measured at 405 nm and the enzyme-activity was calculated after plotting a standard curve. The amylase activity was expressed as Units/L of plasma.

The concentration of pro-inflammatory cytokines was measured in plasma by ELISA- IL-6 (Cat. No. DY406-05, DuoSet, USA), TNF-α (Cat. No. DY410-05, DuoSet, USA), MCP-1 (Cat. No. DY479-05, DuoSet, USA) and GM-CSF (Cat. No. DY415-05, DuoSet, USA) according to manufacturer’s instructions. The concentrations were expressed as pg/mL of plasma.

### Statistical analysis

All data are expressed as the mean ± standard error of mean (SEM) across the repeated experiments. Normally distributed data were compared by one-way Analysis of Variance (ANOVA) and Bonferroni’s *post hoc* correction. Skewed data were compared by Kruskall-Wallis test and *post hoc* Dunn’s test. A p-value <0.05 was considered as significant. All statistical analyses were carried out on SPSS software (version 23.0, IBM, USA).

## Results

### Effect of physiological induction of autophagy by calorie restriction on AP

The blood glucose measured showed that none of the mice was hypoglycemic (<70mg/dL) (31, 32) at any time point, in any of the groups (**Table 1**). There was no significant difference between CRCeAP and NFeCeAP at anytime point (5:30 PM, 7 AM and 5:30 PM; p=0.42, 0.24, 0.69, respectively).

**Table 1:** Blood glucose level (mg/dL) measured at various time points in the four experimental groups (n=6 each).

	Blood glucose level (mg/dL) at various time points		
	5.30 PM (Baseline)Median (Q1-Q3)	7.00 AM (30 min prior to 1^st^ injection)Median (Q1- Q3)	5.30 PM (at time of sacrifice)Median (Q1- Q3)
**CR Control**	135.5 (112.5-151.7)	104.5 (99.0-129.2)	73.5 (65.0-108.0)
**NFe Control**	171.5 (148.5-174.2)	138.5 (129.5-146.5)	96.5 (92.2-106.2)
**CRCeAP**	158.5 (110.2-172.2)	126.5 (115.5-130.7)	75.5 (60.0-84.2)
**NFeCeAP**	151.0 (143.2-169.5)	134.0 (120.5-153.0)	80.0 (57.7-118.2)

Values are represented as median (first quartile-third quartile).

### Status of autophagy in caerulein-induced severe AP with calorie restriction

**Figure 1:**
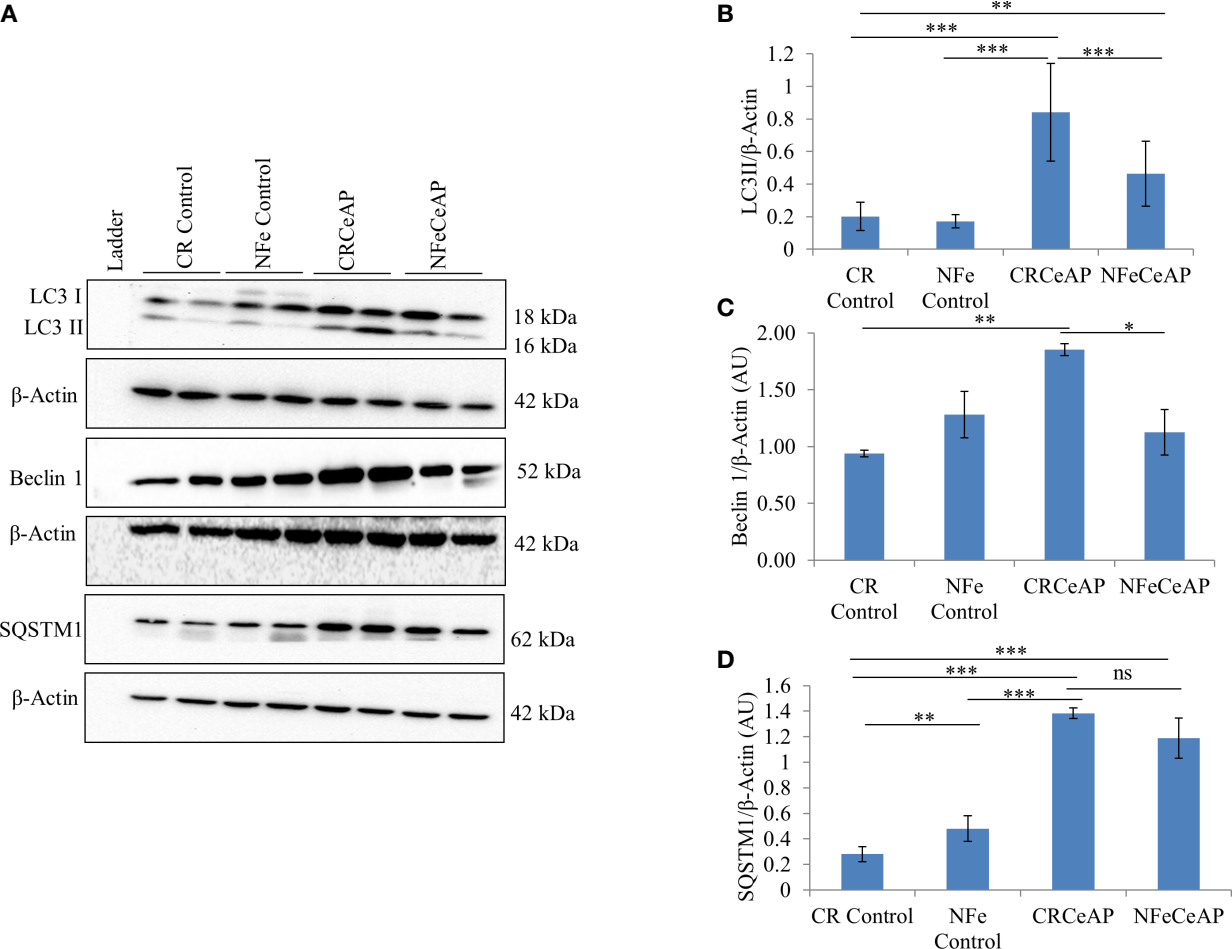
**(A)** Immunoblots for the relative expression of LC3II, Beclin 1 and SQSTM1 in mouse pancreas. β-actin was used to normalize the relative expression of LC3II, Beclin 1 and SQSTM1. **(B–D)**: Bar-diagrams represent mean ± standard error of mean (SEM) of at least 2 separate experiments (n=3 in each experiment). **(B)**: *** = CRCeAP versus NFe Control, p<0.0001; *** = CRCeAP versus NFeCeAP, p<0.0001; ** = NFeCeAP versus CR Control, p=0.001; *** = CRCeAP versus CR Control, p<0.0001 **(C)**: ** = CRCeAP versus CR Control, p= 0.001; * = CRCeAP versus NFeCeAP, p= 0.008 **(D)**: **= NFe Control versus CR Control, p=0.001; *** = CRCeAP versus CR Control, p<0.0001; *** = NFeCeAP versus CR Control, p<0.0001; *** = CRCeAP versus NFe Control, p<0.0001; ns = NFeCeAP versus CRCeAP, p=0.24.

The expression of LC3II was significantly greater in CRCeAP in comparison to NFeCeAP (p<0.0001) (**Figures 1A, B**) implying greater induction of autophagy with calorie-restriction. The expression of Beclin1 was also significantly higher in CRCeAP in comparison to NFeCeAP (p=0.008) (**Figures 1A, C**). Both of these indicated that there was more induction of autophagy in the animals that were fasted overnight. There was no significant difference in the expression of SQSTM1 between CRCeAP and NFeCeAP groups, (p=0.24) (**Figures 1A, D**) implying that caerulein-induced AP affected the completion of the autophagic flux independent of the level of induction.

**Figure 2:**
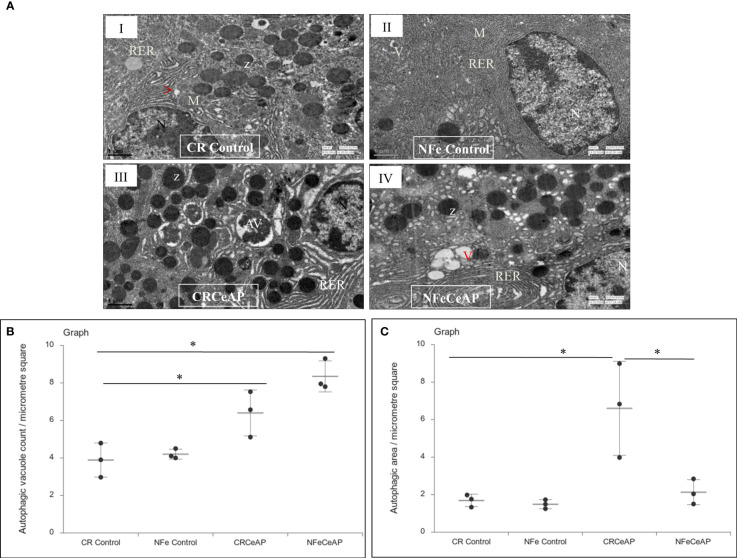
**(A)** Transmission electron micrographs of pancreas derived from mice of: (I) CR control showing normal nucleus (N), rough endoplasmic reticulum (RER), mitochondria (M), zymogen granules (Z) and formation of small autophagic vesicles (arrowhead); (II) NFe control showing zymogen granules, normal nucleus, normal mitochondria, rough endoplasmic reticulum (RER) and small vacuoles (V); (III) CRCeAP group showed large number of autophagic vesicles (AV), large number of zymogen granules and the RER was dilated; (IV) NFeCeAP group showing large number of vacuoles (V) and enlarged ER. Scale bar: I=II=III=IV=1 µm **(B)**: Dot plot representing the mean ± standard deviation (SD) of autophagic vacuole count per micrometer square (n=3). The extent of the vertical bar represents the SD and the transverse bar in the middle is the mean. *= NFeCeAP versus CR Control, p=0.024; *= CRCeAP versus CR Control; p=0.036 **(C)**: Dot plot representing the mean ± SD of percentage of area occupied by vacuole in cytoplasm (n=3). The extent of the vertical bar represents the SD and the transverse bar in the middle is the mean. *= CRCeAP versus CR Control, p=0.05; *= CRCeAP versus NFeCeAP, p=0.032.

The TEM images showed that in the control groups- the nucleus, endoplasmic reticulum and mitochondria were normal (**Figure 2**). There was a higher number of vacuoles in CRCeAP group in comparison to CR Control (p=0.03) (**Figures 2A, B**).The area occupied by vacuoles was also significantly higher in CRCeAP group in comparison to NFeCeAP (p=0.032) (**Figures 2A, C**).Therefore, the completion of autophagy is affected in caerulein-induced severe AP independent of overnight fasting.

### Evaluation of pancreatic tissue injury

**Figure 3:**
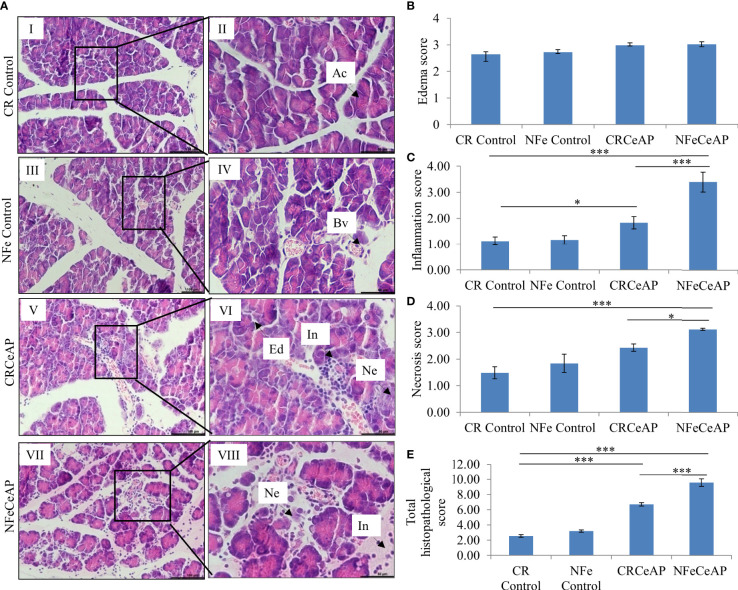
**(A)** Photomicrographs of hematoxylin and eosin stained sections of the pancreas derived from mice of: (I & II) CR control showing normal acini (Ac); (III & IV) NFe Control showing normal acini and blood vessels (Bv); (V & VI) CRCeAP group showing interacinar edema (Ed), necrosis (Ne) and inflammatory infiltration (In); and (VII & VIII) NFeCeAP group which also showed inflammation (In) and necrosis (Ne). Scale bars: I=III=V=VII=100 µm; II=IV=VI=VIII=50 µm **(B)**: Bar-diagram representing edema score as mean ± SEM of at least 2 separate experiments (n=3 in each experiment). **(C)**: Bar-diagram representing inflammation score as mean ± SEM of at least 2 separate experiments (n=3 in each experiment). * = CRCeAP verusus CR Control, p=0.01; *** = NFeCeAP versus CR Control, p<0.0001; ***= CRCeAP versus NFeCeAP, p<0.0001 **(D)**: Bar-diagram representing necrosis score as mean ± SEM of at least 2 separate experiments (n=3 in each experiment). *** = NFeCeAP versus CR Control, p<0.0001; * = CRCeAP versus NFeCeAP, p=0.012 **(E)**: Bar-diagram representing total histopathological pancreatic injury score as mean ± SEM of at least 2 separate experiments (n=3 in each experiment). *** = CRCeAP versus CR Control, p<0.0001; *** = NFeCeAP versus CR Control, p<0.0001; *** = CRCeAP versus NFeCeAP, p<0.0001 *NB: Maximum score of hemorrhage is 4, but during scoring we did not observe any hemorrhage in any high power field in areas; hence, this parameter has been excluded from the tabulation though the total score reported is still 16.*.

Hematoxylin and eosin stained section showed normal acini (Ac) and mild edema in CR Control (**Figure 3AII**). In CRCeAP animal there was interacinar edema, inflammatory infiltration and necrosis (**Figure 3AVI**). NFeCeAP group had more interacinar edema, perivascular inflammation and necrosis in comparison to CRCeAP (p<0.0001) (**Figure 3AVIII**) indicating a greater inflammatory response and irretrievable cell-injury resulting in necrotic cell death. Inflammation and necrosis score were significantly lower in CRCeAP in comparison to NFeCeAP (p<0.0001, p=0.012, respectively) (**Figures 3C, D**). The histopathological score was significantly increased in NFeCeAP versus CRCeAP (p<0.0001) (**Figure 3E**).

**Figure 4:**
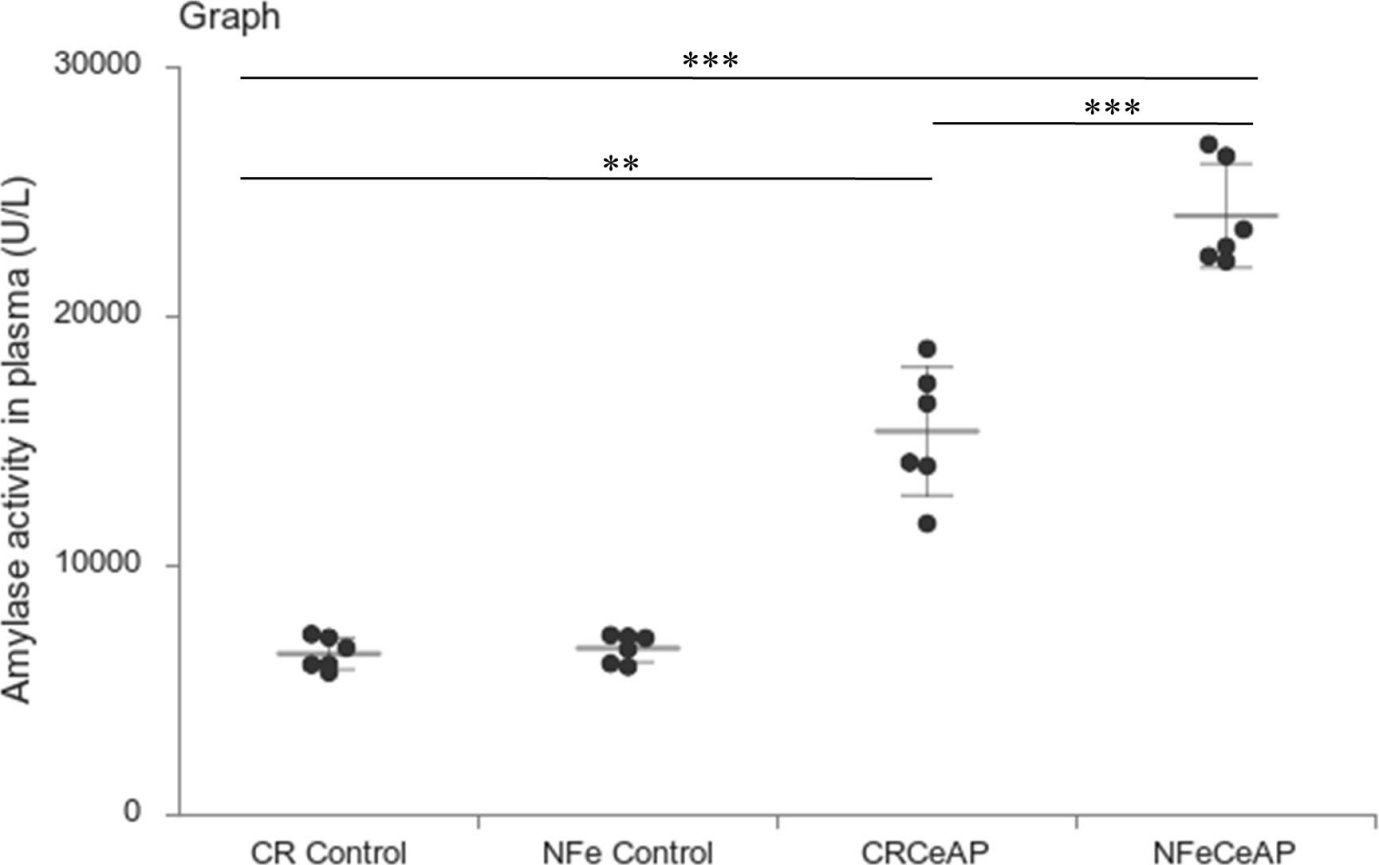
Dot plot representing amylase activity (U/L) in mouse plasma represented as mean ± SD (n=6). The extent of the vertical bar represents the SD and the transverse bar in the middle is the mean. ** = CRCeAP versus CR Control, p=0.001; ***= NFeCeAP versus CR Control, p<0.0001; *** = CRCeAP versus NFeCeAP, p<0.0001.

Amylase activity was significantly higher in the plasma of NFeCeAP in comparison to CRCeAP (p<0.0001) (**Figure 4**).

### Markers of Inflammation and cell death

**Figure 5:**
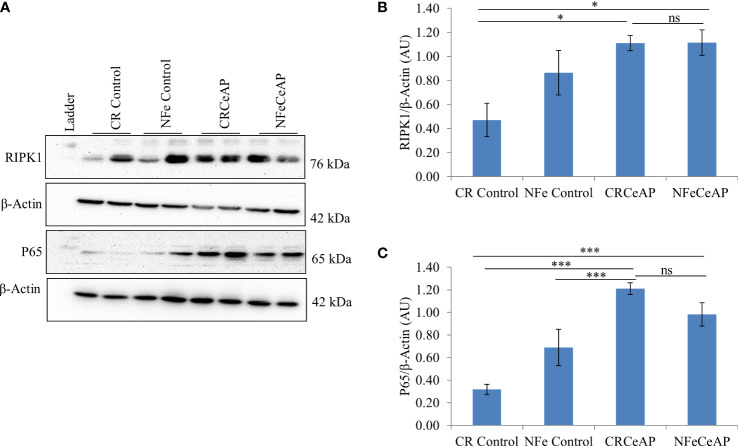
**(A)** Immunoblots for the relative expression of RIPK1 and P65 in mouse pancreas. The relative expression of β-actin was used to normalize the expression of these proteins. **(B, C)**: Bar-diagrams representing the relative expression of each protein to β-actin as mean ± SEM of at least 2 separate experiments (n=3 in each experiment). **(B)**: * = CRCeAP versus CR Control, p=0.024; * = NFeCeAP versus CR Control, p=0.01; ns = NFeCeAP versus CRCeAP, p=1 ; **(C)** *** = CRCeAP versus CR Control, p<0.0001; *** = NFeCeAP versus CR Control, p<0.0001; *** = CRCeAP versus NFe Control, p<0.0001; ns = NFeCeAP versus CRCeAP, p=1.

**Figure 6:**
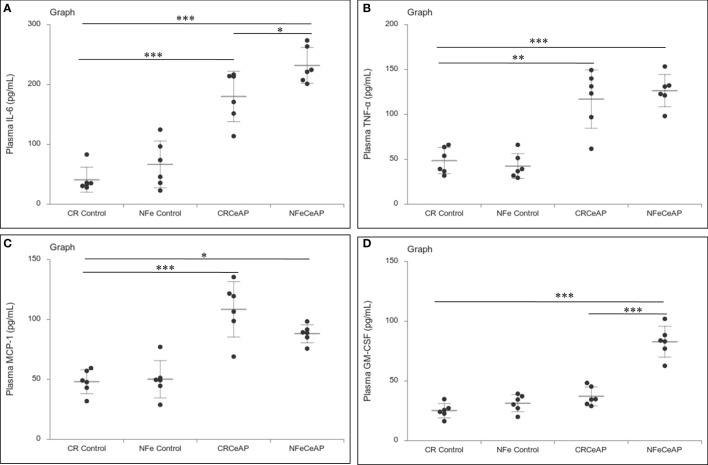
Dot plot representing cytokines level in mouse plasma. The extent of the vertical bar represents the SD and the transverse bar in the middle is the mean. These statistics are derived from at least 2 separate experiments (n=3 in each experiment). **(A)**: IL-6 level in plasma: *** = CRCeAP versus CR Control, p<0.0001; *** = NFeCeAP versus CR Control, p<0.0001; * = NFeCeAP versus CRCeAP, p=0.04 **(B)**: TNF-α level in plasma: ** = CRCeAP versus CR Control, p=0.001; *** = NFeCeAP versus CR Control, p<0.0001 **(C)**: MCP-1 level in plasma: *** = CRCeAP versus CR Control, p<0.0001; * = NFeCeAP versus CR Control, p=0.005 **(D)**: GM-CSF level in plasma: *** = NFeCeAP versus CR Control, p<0.0001; *** = NFeCeAP versus CRCeAP, p<0.0001.

The relative expression of RIPK1 was higher in CRCeAP and NFeCeAP groups (p=0.02, p<0.0001, respectively) in comparison to control (**Figures 5A, B**). The expression of P65 was higher in CRCeAP and NFeCeAP (p<0.0001, p<0.0001, respectively) in comparison to controls (**Figures 5A, C**). Thus, the components of the inflammasome were markedly raised in AP. The concentration of TNF-α and MCP-1 in plasma were higher in CRCeAP and NFeCeAP in comparison to control (p=0.001, p<0.0001, p<0.0001, p=0.005, respectively) (**Figures 6B, C**) as expected in AP. The concentration of IL-6 and GM-CSF in plasma was lower in CRCeAP in comparison to NFeCeAP (p<0.0001, p<0.001, respectively) (**Figures 6A, D**) which corroborated the lower histopathological score of inflammatory infiltration in CRCeAP in comparison to NFeCeAP.

**Figure 7:**
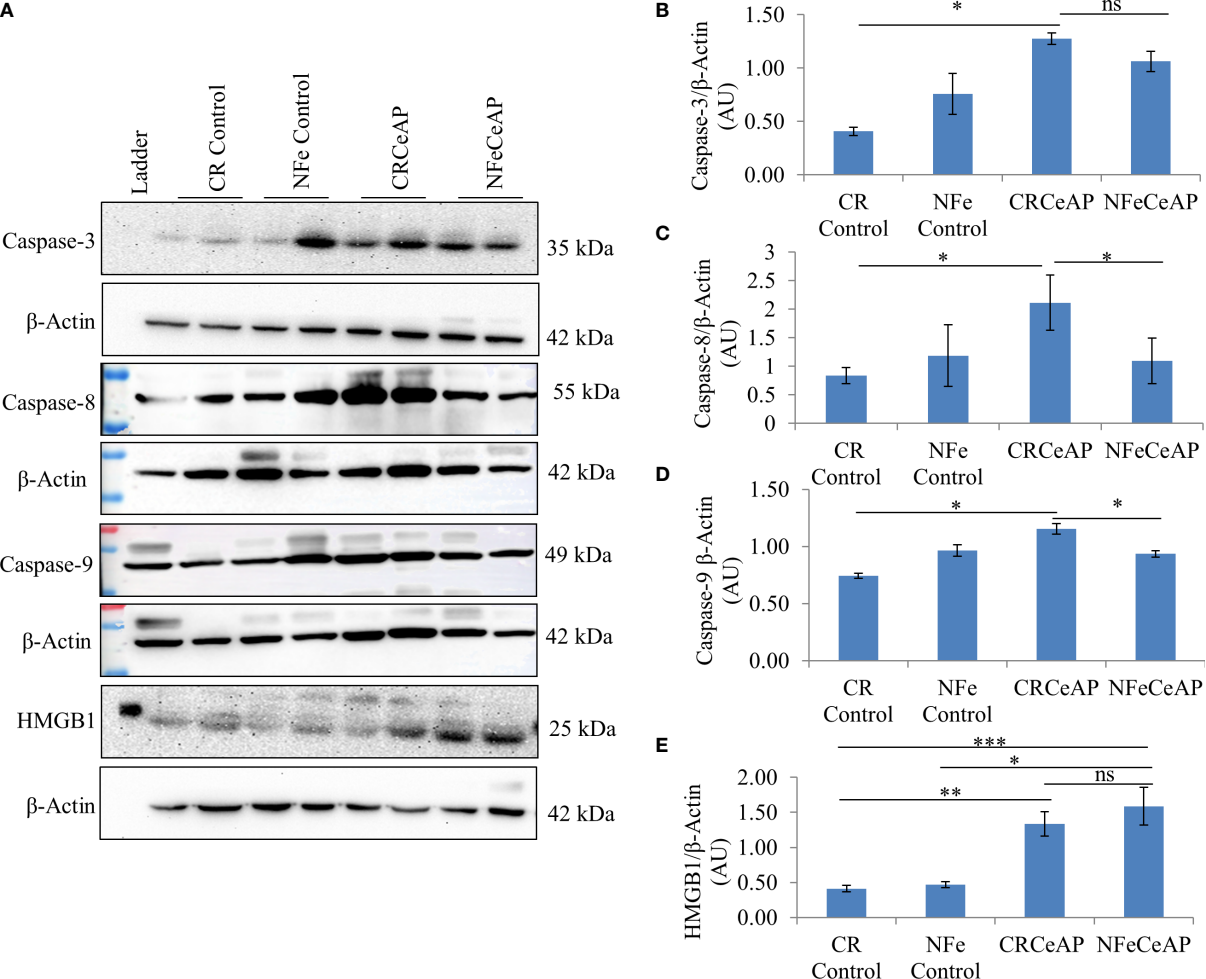
**(A)** Immunoblots for the relative expression of Caspase-3, Caspase-8, Caspase-9 and HMGB1 in mouse pancreas. The relative expression of β-actin was used to normalize the expression of these proteins. **(B–E)**: Bar-diagrams representing the relative expression of each protein to β-actin as mean ± SEM of at least 2 separate experiments (n=3 in each experiment). **(B)**: * = CRCeAP versus CR Control, p=0.013; ns = NFeCeAP versus CRCeAP, p=1 **(C)**: * = CRCeAP versus CR Control, p=0.006; * = NFeCeAP versus CRCeAP, p=0.03 **(D)**: * = CRCeAP versus CR Control, p=0.013; * = NFeCeAP versus CRCeAP, p=0.049 **(E)**: ** = CRCeAP versus CR Control, p=0.001; *** = NFeCeAP versus CR Control, p<0.0001; * = NFeCeAP versus NFe Control, p=0.002; ns = NFeCeAP versus CRCeAP, p=0.38.

The relative expression of caspase-3 was higher in CRCeAP in comparison to control (p=0.013). There was no change in the expression of caspase-3 between NFeCeAP and CRCeAP (p=1) (**Figures 7A, B**).The relative expression of caspase-8 (marker of extrinsic pathway of apoptosis) and caspase-9 (marker of intrinsic pathway of apoptosis) was significantly higher in CRCeAP in comparison to NFeCeAP (p=0.03, p=0.049, respectively) (**Figures 7A, C, D**). The relative expression of HMGB1 was higher in NFeCeAP in comparison to controls (p<0.0001). There was no significant difference in the expression of HMGB1 in NFeCeAP versus CRCeAP (p=0.38) (**Figures 7A, E**). Thus, there was significantly more apoptosis, both by the intrinsic and extrinsic pathways, in CRCeAP in comparison to NFeCeAP.

### Effect of pharmacological induction of autophagy by rapamycin on seve*re AP*

#### Status of autophagy in caerulein-induced severe AP pre-treated with rapamycin

**Figure 8:**
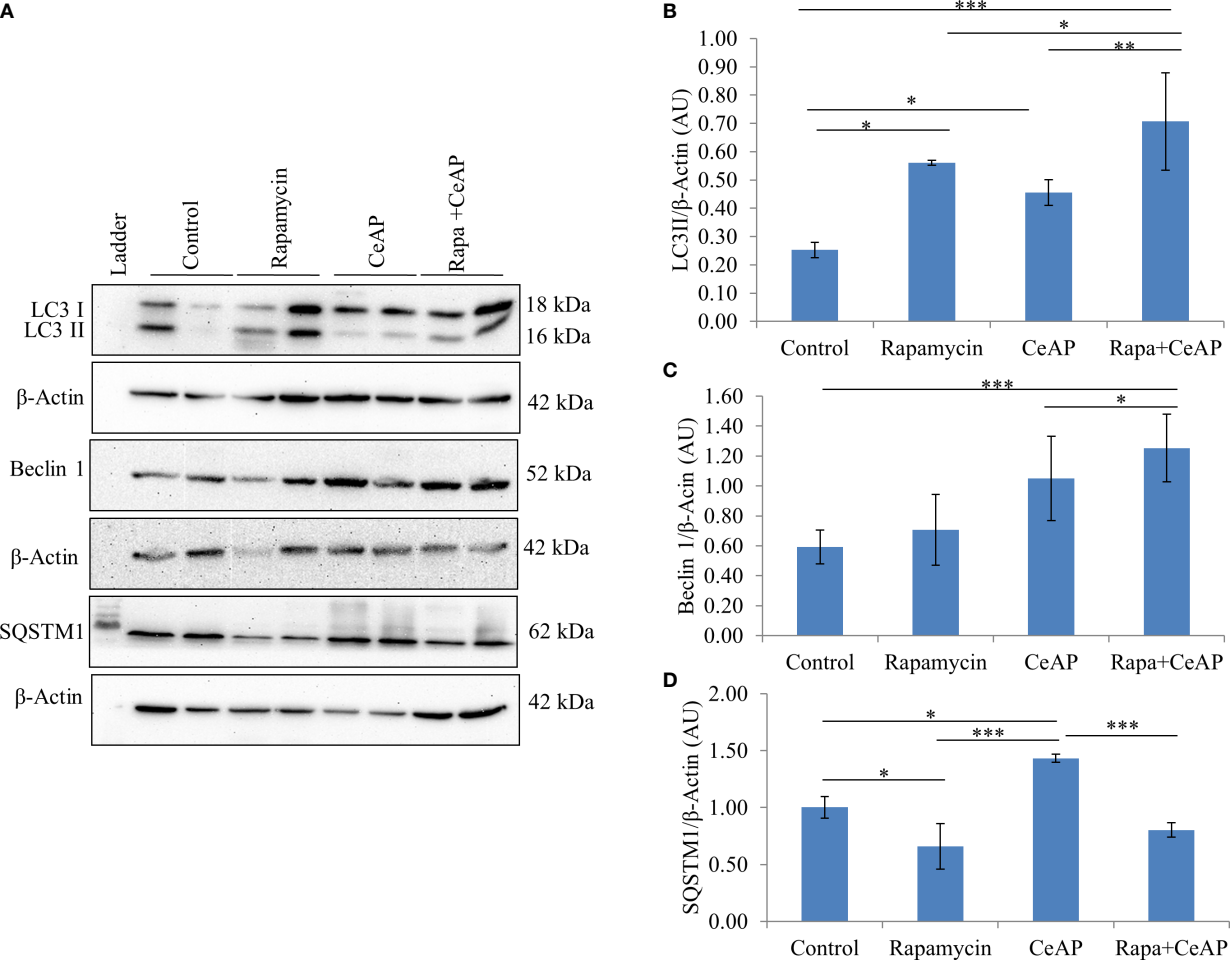
**(A)** Immunoblots for the relative expression of LC3II, Beclin 1 and SQSTM1 in mouse pancreas. β-actin was used to normalize the relative expression of LC3II Beclin 1 and SQSTM1. **(B–D)**: Bar-diagrams represent mean ± SEM of at least 2 separate experiments (n=3 in each experiment). **(B)**: * = Control versus Rapamycin, p=0.05; * = CeAP versus Control, p=0.029; *** = Rapa+CeAP versus Control, p<0.0001; * = Rapa+CeAP versus Rapamycin, p=0.041; ** = Rapa+CeAP versus CeAP, p=0.001 **(C)**: *** = Rapa+CeAP versus Control, p<0.0001; * = Rapa+CeAP versus CeAP, p=0.005 **(D)**: * = Control versus Rapamycin, p=0.01; * = CeAP versus Control, p=0.05; *** = CeAP versus Rapamycin, p<0.0001; *** = Rapa+CeAP versus CeAP, p<0.0001.

**Figure 9:**
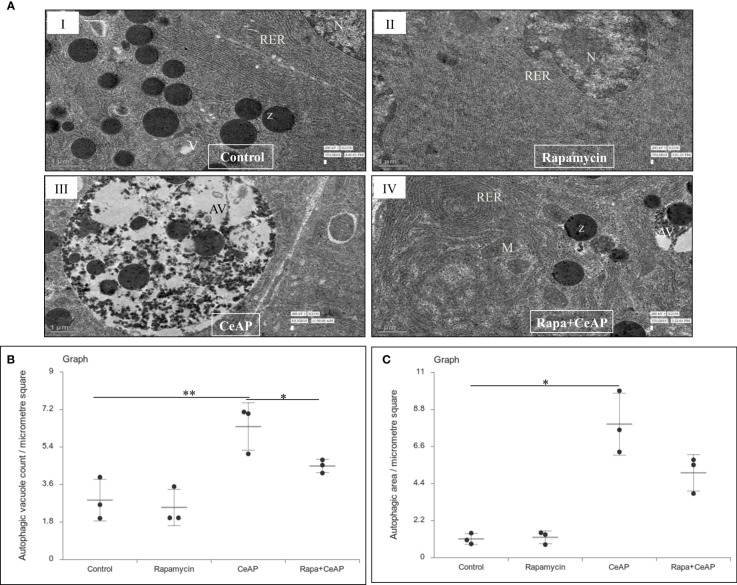
**(A)** Transmission electron micrographs of pancreas derived from mice of (I) control showing normal nucleus (N), rough endoplasmic reticulum (RER), zymogen granules (Z) and vacuoles (V); (II) Rapamycin control showing normal nucleus (N) and endoplasmic reticulum (ER); (III) CeAP group showing large autophagic vesicles (AV); (IV) Rapa+CeAP group showing normal rough endoplasmic reticulum (RER), mitochondria (M), zymogen granules (Z) and small autophagic vacuoles (AV). Scale bar: I=II=III=IV=1 µm **(B)**: Dot plot representing the mean ± SD of autophagic vacuole count per micrometer square (n=3). The extent of the vertical bar represents the SD and the transverse bar in the middle is the mean. ** = CeAP versus Control, p=0.001; * **=** Rapa+CeAP versus CeAP, p=0.004 **(C)**: Dot plot representing the mean ± SD of percentage of area occupied by vacuole in cytoplasm (n=3). The extent of the vertical bar represents the SD and the transverse bar in the middle is the mean. * = CeAP versus Control, p=0.048.

The relative expression of LC3II and Beclin 1 were significantly higher in Rapa+CeAP when compared to the CeAP (p=0.001, p=0.005, respectively) (**Figures 8A–C**). The relative expression of SQSTM1 was significantly lower in Rapa+CeAP when compared to CeAP (p<0.0001) (**Figures 8A, D**). Vacuole number and vacuole area occupied in cytoplasm were also higher in CeAP in comparison to control (p=0.001, p=0.048, respectively) (**Figure 9**). The number of vacuoles in Rapa+CeAP was significantly lower in comparison to CeAP (p=0.004) (**Figure 9B**). Hence, rapamycin not only increased the induction of autophagy but also helped in its completion even in the presence of caerulein-induced AP.

#### Evaluation of pancreatic tissue injury

**Figure 10:**
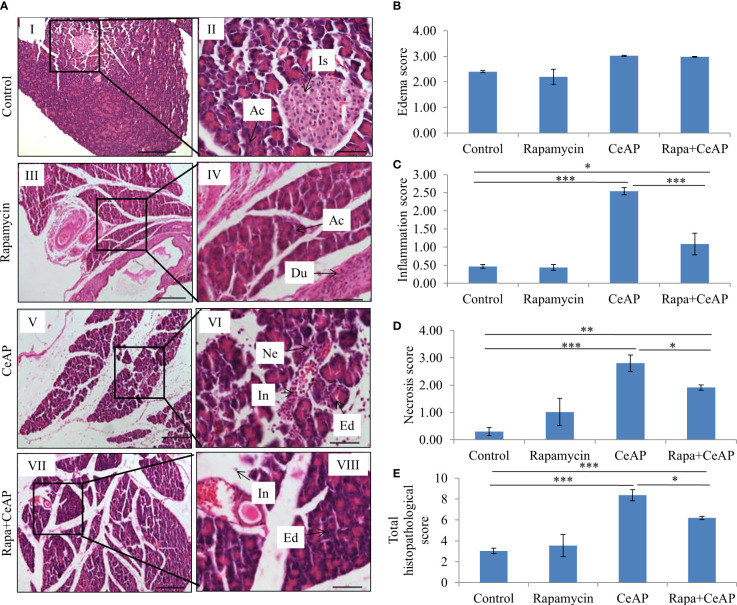
**(A)** Photomicrographs of hematoxylin and eosin stained sections of the pancreas derived from mice of (I & II) control showing normal acini (Ac) and islet (Is); (III & IV) Rapamycin control showing normal acini (Ac) and duct (Du); (V & VI) was given 8, hourly doses of caerulein (CeAP). The pancreas show interacinar edema (Ed), necrosis (Ne) and inflammatory infiltration (In); and (VII & VIII) Rapa+CeAP group showing edema (Ed) and few inflammatory cells (In). Scale bars: I=III=V=VII=200 µm; II=IV=VI=VIII=50 µm **(B)**: Bar-diagram representing edema score as mean ± SEM of at least 2 separate experiments (n=3 in each experiment). **(C)**: Bar-diagram representing inflammation score as mean ± SEM of at least 2 separate experiments (n=3 in each experiment). *** = CeAP versus Control, p<0.0001; * = Rapa+CeAP versus Control, p=0.012; *** = Rapa+CeAP versus CeAP, p<0.0001 **(D)**: Bar-diagram representing necrosis score as mean ± SEM of at least 2 separate experiments (n=3 in each experiment). *** = CeAP versus Control, p<0.0001; ** = Rapa+CeAP versus Control, p=0.001; * = Rapa+CeAP versus CeAP, p=0.01 **(E)**: Bar-diagram representing total histopathological pancreatic injury score as mean ± SEM of at least 2 separate experiments (n=3 in each experiment). *** = CeAP versus Control, p<0.0001; *** = Rapa+CeAP versus Control, p<0.0001; * = Rapa+CeAP versus CeAP, p=0.004 *NB: Maximum score of hemorrhage is 4, but during scoring we did not observe any hemorrhage in any high power field in areas; hence, this parameter has been excluded from the tabulation though the total score reported is still 16.*.

**Figure 11:**
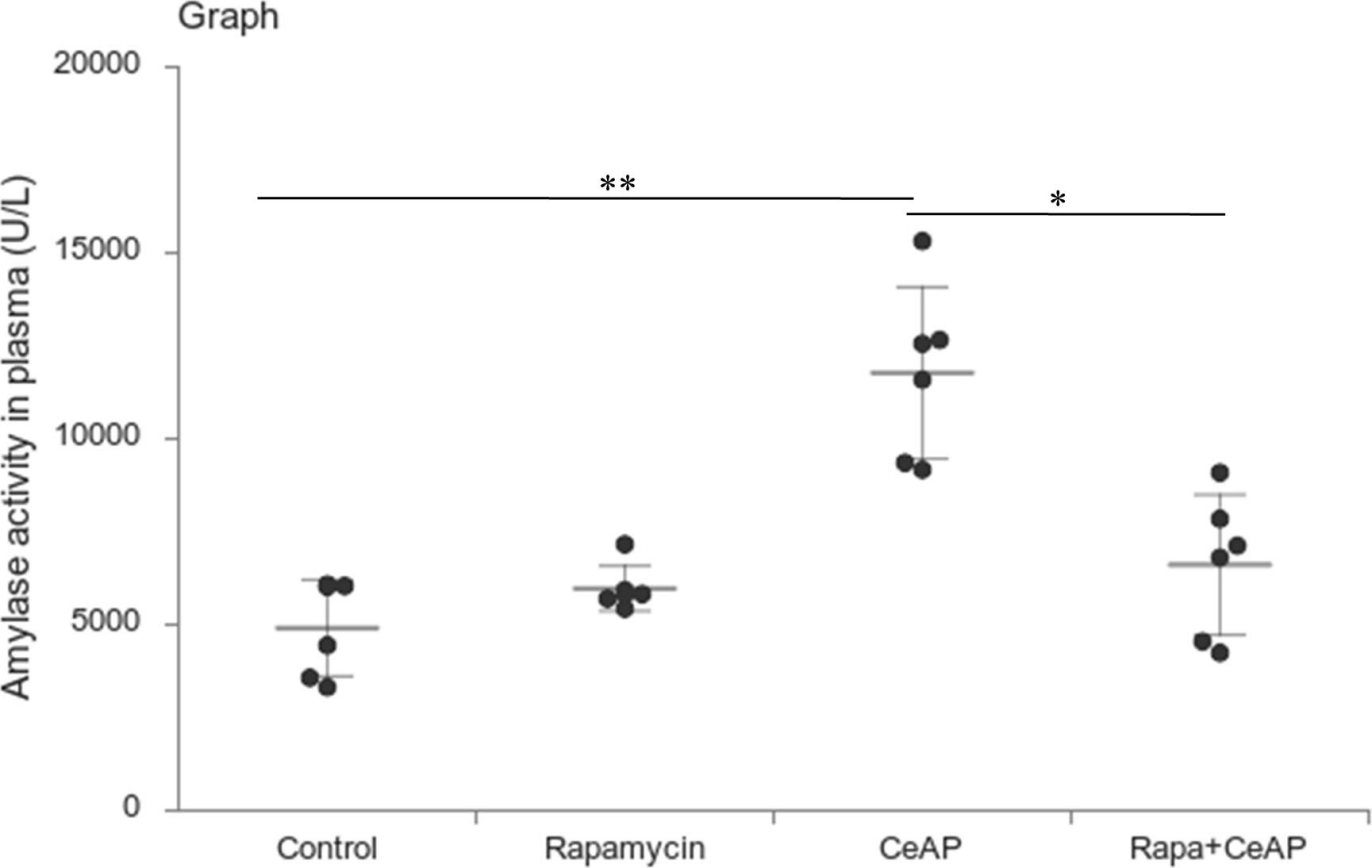
Dot plot representing amylase activity (U/L) in mouse plasma represented as mean ± SD (n=6). The extent of the vertical bar represents the SD and the transverse bar in the middle is the mean. ** = CeAP versus Control, p=0.001; * = Rapa+CeAP versus CeAP, p=0.021.

Rapamycin control animals showed normal pancreatic architecture (**Figure 10AII**). Rapa+CeAP had interacinar edema and fewer inflammatory cells in comparison to CeAP (**Figure 10AVIII**). Both inflammation and necrosis were significantly lower in Rapa+CeAP in comparison to CeAP (p<0.0001, p=0.01, respectively) (**Figures 10C, D**). The histopathological score was significantly lower in Rapa+CeAP compared with CeAP (p=0.004) (**Figure 10E**). Amylase activity was also significantly lesser in plasma of Rapa+CeAP when compared to CeAP (p=0.021) (**Figure 11**).

#### Inflammation and mode of cell death

**Figure 12:**
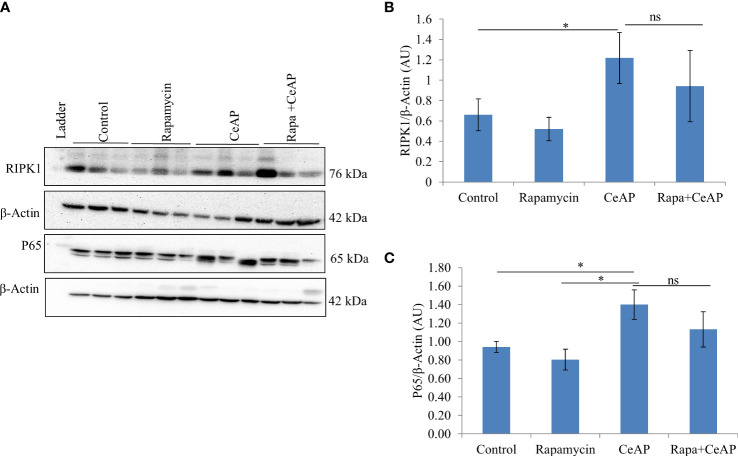
**(A)** Immunoblots for the relative expression of RIPK1 and P65 in mouse pancreas. The relative expression of β-actin was used to normalize the expression of these proteins. **(B, C)**: Bar-diagrams representing the relative expression of each protein to β-actin as mean ± SEM of at least 2 separate experiments (n=3 in each experiment). **(B)**: * = CeAP versus Control, p=0.05; ns = Rapa+CeAP versus CeAP, p=1 **(C)**: * = CeAP versus Rapamycin, p=0.041; * = CeAP versus Control, p=0.045; ns = Rapa+CeAP versus CeAP, p=1.

**Figure 13:**
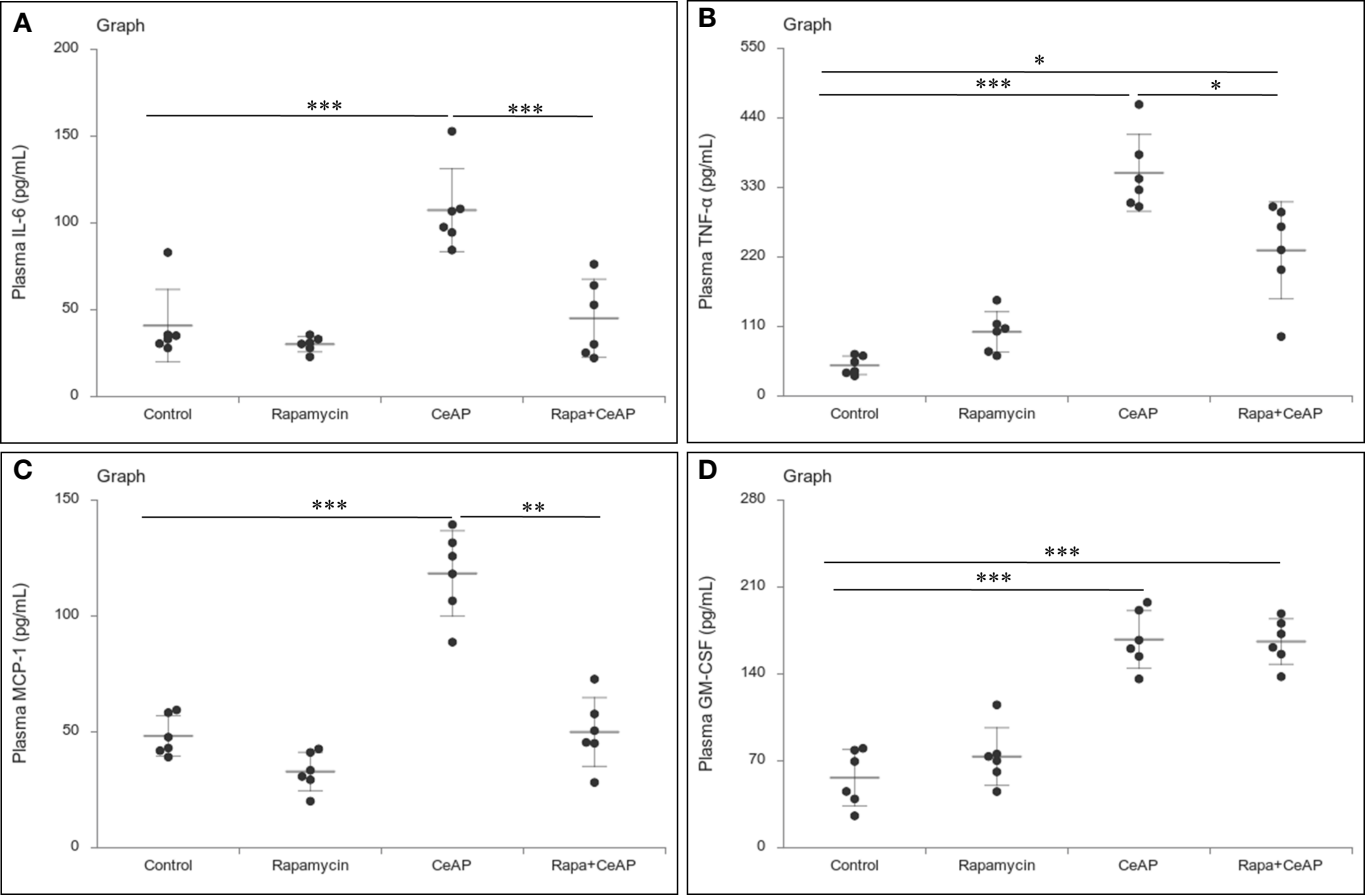
Dot plot representing cytokines level in mouse plasma. The extent of the vertical bar represents the SD and the transverse bar in the middle is the mean. These statistics are derived from at least 2 separate experiments (n=3 in each experiment). **(A)**: IL-6 level in plasma: *** = CeAP versus Control, p<0.0001; *** = Rapa+CeAP versus CeAP, p<0.0001 **(B)**: TNF-α level in plasma: *** = CeAP versus Control, p<0.0001, * = Rapa+CeAP versus Control, p=0.0046; * = Rapa+CeAP versus CeAP, p=0.047 **(C)**: MCP-1 level in plasma: *** = CeAP versus Control, p<0.0001; ** = Rapa+CeAP versus CeAP, p=0.001 **(D)**: GM-CSF level in plasma: *** = CeAP versus Control, p<0.0001; *** = Rapa+CeAP versus Control, p<0.0001.

The relative expression of RIPK1 was similar in Rapa+CeAP and CeAP (p=1) (**Figures 12A, B**). The relative expression of P65 was higher in CeAP in comparison to control (p=0.045) (**Figures 12A, C**). The concentration of IL-6, TNF-α and MCP-1 in plasma was lower in Rapa+CeAP in comparison to CeAP (p<0.0001, p=0.0046, p=0.001, respectively) (**Figures 13A–C**) which supported the lower histopathological inflammatory score seen in Rapa+CeAP. There was no change in the level of GM-CSF between Rapa+CeAP and CeAP (**Figure 13D**).

**Figure 14:**
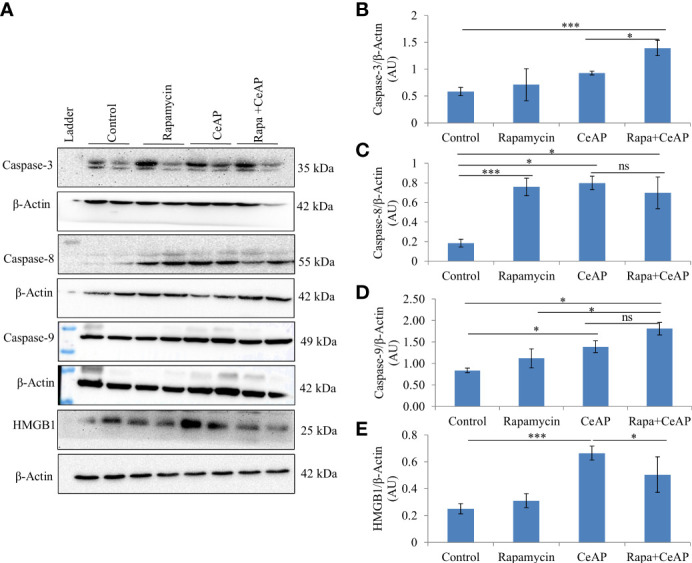
**(A)** Immunoblots for the relative expression of Caspase-3, Caspase-8, Caspase-9 and HMGB1 in mouse pancreas. The relative expression of β-actin was used to normalize the expression of these proteins. **(B–E)**: Bar-diagrams representing the relative expression of each protein to β-actin as mean ± SEM of at least 2 separate experiments (n=3 in each experiment). **(B)**: *** = Rapa+CeAP versus Control, p<0.0001; * = Rapa+CeAP versus CeAP, p=0.016 **(C)**: *** = Rapamycin versus Control, p<0.0001;* = CeAP versus Control, p=0.03; * = Rapa+CeAP versus Control, p=0.008; ns= Rapa+CeAP versus CeAP, p=1 **(D)**: * = CeAP versus Control, p=0.044; *=Rapa+CeAP versus Rapamycin, p=0.048; * = Rapa+CeAP versus Control, p=0.012; ns= Rapa+CeAP versus CeAP, p=0.2 **(E)**: *** = CeAP versus Control, p<0.0001; * = Rapa+CeAP versus CeAP, p=0.011.

The relative expression of caspase-3 was more in Rapa+CeAP when compared to CeAP (p=0.016) **Figures 14A, B**), implying that apoptotic cell death was promoted in the injured pancreatic cell by the administration of rapamycin. The relative expression of caspase-8 and caspase-9 was also higher in CeAP and Rapa+CeAP in comparison to controls (p=0.03, p=0.008, p=0.044, p=0.012, respectively) (**Figures 14A–D**). The relative expression of HMGB1 was lower in Rapa+CeAP when compared to CeAP (p=0.011) **Figures 14 A, E**); implying that rapamycin led to significantly lesser necrosis.

## Discussion

In the present study, we found that in the caerulein-induced model of AP, the induction of autophagy by either calorie-restriction or rapamycin decreased the severity of the disease by enhancing the autophagic flux, reducing necrotic cell death and reducing inflammation.

Prolonged restriction of calories did not lead to hypoglycemia or induction of autophagy in the control mice in the present study (**Table 1**). After inducing pancreatitis, mice in the calorie restriction had greater induction of autophagy (**Figure 1**). Hence, the mechanism of greater induction of autophagy in this group may be independent of blood glucose level and may be due to other factors. This scenario of overnight fasting is similar to the clinical setting of preparing a patient for endoscopic retrograde cholangiopancreatography (ERCP) and this may also be the reason why post-ERCP pancreatitis is uncommon and is usually mild (33).

The completion of the autophagic flux is marked by the digestion of the protein p62 or SQSTM1, which is an integral part of the inner membrane of the autophagic vacuole (34). When the autophagic vacuole fuses with lysosomes, the proteins in the wall of the phagophore are also lysed; hence, the reduction in the elevated levels of SQSTM1 is an indication of completion of the autophagic flux (8, 35). As expected and shown previously (8) caerulein, used at a dose to induce severe AP, blocks the autophagic flux and leads to greater cell necrosis and inflammation (36, 37). In our experiments, we too noted that the completion of autophagic flux was blocked in severe experimental AP (**Figures 1A, D**). But we observed something significant. One of the premises of this study is that calorie-restriction can induce autophagy and this is restricted by providing the mice free access to chow. Hence, mice in the NFeCeAP also had a lower baseline induction of autophagy and this may be the reason why they also demonstrated a lower total area occupied by autophagosomes (**Figure 2**). Here, the induction of autophagy is induced by the caerulein-induced pancreatitis, which also blocks the autophagic flux. Hence, the protein markers of the induction of autophagy (Beclin1 and LC3II) are higher in CRCeAP and that of completion of autophagy (SQSTM1) was equivalent in both groups. We may conclude that calorie-restriction allows better completion of the autophagic flux.

However, the severity of AP was significantly lesser in the mice that were in calorie restriction group, which was primarily due to reduction in inflammation (**Figure 3**). This may be because calorie-restriction has been shown to reduce the expression of IL-6 receptors (38), and IL-6 is known to be a key mediator of the inflammatory response in AP (39). In addition, neutrophils function best in optimum levels of glucose; it is likely that the low levels of blood sugar may have reduced the motility and degranulation response of neutrophils (40). Further, the increased inflammation and plasma concentration of IL-6 seen in severe AP is also known to cause the phosphorylation of the regulator protein STAT3, which in turn induces increased levels of Bcl-2, reduces Beclin1 and thus inhibits autophagy (41). The key molecule that creates the switch between programmed cell death and autophagy is RIPK1 and its ubiquitination (42). Increased ubiquitination of RIPK1 decreases its expression. This decreases its ability to release NF-kB from its inhibitory complex which decreases the production of inflammatory mediators and the apoptotic cascade. When this ubiquitination is hampered, inflammation increases. Thus, in the absence of this ubiquitination, the injured cell switches to autophagy to attempt recovery (43, 44). Here, we found that calorie restriction did not alter the mode of cell death, but it produced a definitive decrease in the expression of RIPK1 and IL-6, and histological inflammation (**Figures 5**, **6**, **3**).

When we used rapamycin to induce autophagy, we observed enhanced induction of autophagy and also completion of autophagy, higher conversion to LC3-II, increased level of Beclin1 and number of autophagic vesicles, and decreased level of SQSTM1. The latter could be due to rapamycin-induced formation and recruitment of a larger number of lysosomes in the mammalian cells (45). As a consequence of this greater induction and completion of autophagic process, rapamycin treated mice had significantly lesser severity of pancreatitis with lower inflammation and necrosis scores (**Figure 10**). Biochemically too, we found evidence of lower inflammation that was characterized by significantly lower levels of RIPK1 and IL-6 with rapamycin treatment (**Figures 12**, **13A**). The lower inflammation with rapamycin is also likely to be due to greater amount of apoptotic mode of cell death in the injured pancreatic acinar cells than necrosis (46, 47) as was evident by the higher level of caspase-3 and lower levels of HMGB1 (**Figure 14**). A study suggest that rapamycin attenuates the amplification of IL-6, TNF-α and caspase-3 in intra cerebral hemorrhage induced tissue of rat brain (48). However induction of autophagy by rapamycin and its antiapoptotic effect is not clear (49). Autophagy and apoptosis are connected to each other. In majority of cases, autophagy inhibits the apoptotic form of cell death but in some stress conditions like ER stress autophagy and apoptosis can be activated at same time (50). A recent study has also shown that restoring autophagy by inducing it with rapamycin may ameliorate hypertriglyceridemia-induced AP (51).

The limitations of the present study were that: (i) we were unable to clearly determine which factor induced autophagy in the scenario of calorie-restriction, and (ii) we didn’t assess the tissue concentration of rapamycin. However, previously published data have shown adequate tissue penetration for this drug (22, 52, 53).

With regard to the clinical implications of our findings, calorie restriction in the first few days of AP in humans is usually practiced, although continued calorie-restriction maybe harmful. Methods to induce and complete the autophagic flux may be useful in patients with recurrent AP. Calorie restriction might also be clinically relevant in case of post-endoscopic retrograde cholangiopancreatography (ERCP) pancreatitis, an iatrogenic form of AP (54–56). A prolonged period of fasting might be tested in a clinical trial to check whether it leads to either a lower incidence or lesser severity of post-ERCP pancreatitis compared to standard practice.

In summary, we have shown that the enhancement of not only the induction of autophagy but also the completion of its flux by calorie restriction and rapamycin help in significantly reducing the severity of caerulein-induced AP in mice.

## Data availability statement

The original contributions presented in the study are included in the article/**Supplementary Material**. Further inquiries can be directed to the corresponding author.

## Ethics statement

The animal study was reviewed and approved by Institutional Animal Ethics Committee, All India Institute of Medical Sciences, New Delhi (941/IAEC/16).

## Author contributions

MS executed the study, analyzed the results and wrote the first draft of the manuscript. KP and PK helped in the execution of the study. PG and TR helped in the design of the study, analysis of the results, edited and approved the manuscript. TJ designed the study, analyzed the results, edited and approved the final manuscript. All authors approved the final manuscript prior to submission.

## Funding

This work was supported by Indian Council of Medical Research (ICMR), Government of India as funding of MS (3/1/3/JRF-2013/HRD-120).

## Acknowledgments

The authors of the manuscript would like to acknowledge the support of the Sophisticated Analytical Instrumentation Facility for electron microscopy, and the Central Animal Facility, All India Institute of Medical Sciences, New Delhi. Amylase assay was carried out under the guidance of Dr. Sudip Kumar Datta at the department of Laboratory Medicine, All India Institute of Medical Sciences, New Delhi.

## Conflict of interest

The authors declare that the research was conducted in the absence of any commercial or financial relationships that could be construed as a potential conflict of interest.

## Publisher’s note

All claims expressed in this article are solely those of the authors and do not necessarily represent those of their affiliated organizations, or those of the publisher, the editors and the reviewers. Any product that may be evaluated in this article, or claim that may be made by its manufacturer, is not guaranteed or endorsed by the publisher.

## Abbreviations

AP, acute pancreatitis; CR, calorie restriction; CeAP, caerulein induced AP; CRCeAP, calorie restricted caerulein induced AP; HMGB1, high mobility group box 1; IL-6, interleukine-6; GM-CSF, granulocyte-macrophage colony- stimulating factor; LC3, microtubule associated proteins 1A/1B light chain 3; MCP-1, monocyte chemoattractant protein-1; NFe, night feeding; NFeCeAP, night feeding with CeAP; RIPK1, receptor interacting protein kinase 1; Rapa+CeAP, rapamycin with caerulein induced AP.

## References

[1] Petrov MS Yadav D. Global epidemiology and holistic prevention of pancreatitis. Nat Rev Gastroenterol Hepatol (2019) 16(3):175–84. doi: 10.1038/s41575-018-0087-5 PMC6597260 30482911

[2] Jagannath S Garg PK. Recurrent acute pancreatitis: current concepts in the diagnosis and management. Curr Treat options Gastroenterol (2018) 16(4):449–65. doi: 10.1007/s11938-018-0196-9 30232693

[3] Sah RP Garg P Saluja AK. Pathogenic mechanisms of acute pancreatitis. Curr Opin Gastroenterol (2012) 28(5):507–15. doi: 10.1097/MOG.0b013e3283567f52 PMC3682674 22885948

[4] Jacob TG Raghav R Kumar A Garg PK Roy TS. Duration of injury correlates with necrosis in caerulein-induced experimental acute pancreatitis: implications for pathophysiology. Int J Exp Pathol (2014) 95(3):199–208. doi: 10.1111/iep.12081 24761825 PMC4351856

[5] Barrera K Stanek A Okochi K Niewiadomska Z Mueller C Ou P. Acinar cell injury induced by inadequate unfolded protein response in acute pancreatitis. World J Gastrointest Pathophysiol (2018) 9(2):37–46. doi: 10.4291/wjgp.v9.i2.37 30283709 PMC6163129

[6] Szmola R Sahin-Tóth M. Pancreatitis-associated chymotrypsinogen c (CTRC) mutant elicits endoplasmic reticulum stress in pancreatic acinar cells. Gut. (2010) 59(3):365–72. doi: 10.1136/gut.2009.198903 PMC2848392 19951900

[7] Biczo G Vegh ET Shalbueva N Mareninova OA Elperin J Lotshaw E. Mitochondrial dysfunction, through impaired autophagy, leads to endoplasmic reticulum stress, deregulated lipid metabolism, and pancreatitis in animal models. Gastroenterology. (2018) 154(3):689–703. doi: 10.1053/j.gastro.2017.10.012 29074451 PMC6369139

[8] Yoshii SR Mizushima N. Monitoring and measuring autophagy. Int J Mol Sci (2017) 18(9):1865. doi: 10.3390/ijms18091865 28846632 PMC5618514

[9] Levine B Kroemer G. Autophagy in the pathogenesis of disease. Cell. (2008) 132(1):27–42. doi: 10.1016/j.cell.2007.12.018 18191218 PMC2696814

[10] Kroemer G Mariño G Levine B. Autophagy and the integrated stress response. Mol Cell (2010) 40(2):280–93. doi: 10.1016/j.molcel.2010.09.023 PMC3127250 20965422

[11] Murrow L Debnath J. Autophagy as a stress-response and quality-control mechanism: implications for cell injury and human disease. Annu Rev Pathol Mech Dis (2013) 8(1):105–37. doi: 10.1146/annurev-pathol-020712-163918 PMC3971121 23072311

[12] Gukovskaya AS Gukovsky I. Autophagy and pancreatitis. Am J Physiol - Gastrointest Liver Physiol (2012) 303(9):G993–1003. doi: 10.1152/ajpgi.00122.2012 22961802 PMC3517664

[13] Jensen T Kiersgaard M Sørensen D Mikkelsen L. Fasting of mice: a review. Lab Anim (2013) 47(4):225–40. doi: 10.1177/0023677213501659 24025567

[14] Park JM Lee S Chung MK Kwon S-H Kim E-H Ko KH. Antioxidative phytoceuticals to ameliorate pancreatitis in animal models: An answer from nature. World J Gastroenterol (2014) 20(44):16570. doi: 10.3748/wjg.v20.i44.16570 25469025 PMC4248200

[15] Ding SP Li J-C Jin C. A mouse model of severe acute pancreatitis induced with caerulein and lipopolysaccharide. World J Gastroenterol (2003) 9(3):584. doi: 10.3748/wjg.v9.i3.584 12632523 PMC4621587

[16] Chung KW Chung HY. The effects of calorie restriction on autophagy: role on aging intervention. Nutrients. (2019) 11(12):2923. doi: 10.3390/nu11122923 31810345 PMC6950580

[17] Benjamin D Colombi M Moroni C Hall MN. Rapamycin passes the torch: a new generation of mTOR inhibitors. Nat Rev Drug Discovery (2011) 10(11):868–80. doi: 10.1038/nrd3531 22037041

[18] Antonucci L Fagman JB Kim JY Todoric J Gukovsky I Mackey M. Basal autophagy maintains pancreatic acinar cell homeostasis and protein synthesis and prevents ER stress. Proc Natl Acad Sci (2015) 112(45):E6166–74. doi: 10.1073/pnas.1519384112 PMC4653219 26512112

[19] Hashimoto D Ohmuraya M Hirota M Yamamoto A Suyama K Ida S. Involvement of autophagy in trypsinogen activation within the pancreatic acinar cells. J Cell Biol (2008) 181(7):1065–72. doi: 10.1083/jcb.200712156 PMC2442206 18591426

[20] Ballou LM Lin RZ. Rapamycin and mTOR kinase inhibitors. J Chem Biol (2008) 1(1–4):27–36. doi: 10.1007/s12154-008-0003-5 19568796 PMC2698317

[21] Lin X Han L Weng J Wang K Chen T. Rapamycin inhibits proliferation and induces autophagy in human neuroblastoma cells. Biosci Rep (2018) 38(6):BSR20181822. doi: 10.1042/BSR20181822 30393233 PMC6265625

[22] Wan J Chen J Wu D Yang X Ouyang Y Zhu Y. Regulation of autophagy affects the prognosis of mice with severe acute pancreatitis. Dig Dis Sci (2018) 63(10):2639–50. doi: 10.1007/s10620-018-5053-0 29629491

[23] Schmidt J Rattner DW Lewandrowski K Compton CC Mandavilli U Knoefel WT. A better model of acute pancreatitis for evaluating therapy. Ann Surg (1992) 215(1):44–56. doi: 10.1097/00000658-199201000-00007 1731649 PMC1242369

[24] Jacob TG Sreekumar VI Roy TS Garg PK. Electron-microscopic evidence of mitochondriae containing macroautophagy in experimental acute pancreatitis: Implications for cell death. Pancreatology. (2014) 14(6):454–8. doi: 10.1016/j.pan.2014.08.009 25280593

[25] Mirra S García-Arroyo R B Domènech E Gavaldà-Navarro A Herrera-Úbeda C Oliva C. CERKL, a retinal dystrophy gene, regulates mitochondrial function and dynamics in the mammalian retina. Neurobiol Dis (2021) 156:105405. doi: 10.1016/j.nbd.2021.105405 34048907

[26] Klionsky DJ Abdel-Aziz AK Abdelfatah S Abdellatif M Abdoli A Abel S. Guidelines for the use and interpretation of assays for monitoring autophagy (4th edition) ^1^ . Autophagy. (2021) 17(1):1–382. doi: 10.1080/15548627.2020.1797280 33634751 PMC7996087

[27] Yang Zw Meng Xx Xu P. Central role of neutrophil in the pathogenesis of severe acute pancreatitis. J Cell Mol Med (2015) 19(11):2513–20. doi: 10.1111/jcmm.12639 PMC4627557 26249268

[28] Kearney CJ Martin SJ. An inflammatory perspective on necroptosis. Mol Cell (2017) 65(6):965–73. doi: 10.1016/j.molcel.2017.02.024 28306512

[29] Bedoui S Herold MJ Strasser A. Emerging connectivity of programmed cell death pathways and its physiological implications. Nat Rev Mol Cell Biol (2020) 21(11):678–95. doi: 10.1038/s41580-020-0270-8 32873928

[30] Yu M Wang H Ding A Golenbock DT Latz E Czura CJ. HMGB1 signals through TOLL-like receptor (TLR) 4 and TLR2. Shock (2006) 26(2):174–9. doi: 10.1097/01.shk.0000225404.51320.82 16878026

[31] Sprague JE. Glucose counterregulatory responses to hypoglycemia. Pediatr Endocrinol Rev (2011) 9(1):463–75. PMC3755377 22783644

[32] Sun C Li X Liu L Canet MJ Guan Y Fan Y. Effect of fasting time on measuring mouse blood glucose level. Int J Clin Exp Med (2016) 9(2):4186–9.

[33] Bhatia V Garg PK Tandon RK Madan K. Endoscopic retrograde cholangiopancreatography-induced acute pancreatitis often has a benign outcome. J Clin Gastroenterol (2006) 40(8):726–31. doi: 10.1097/00004836-200609000-00013 16940887

[34] Mizushima N Yoshimori T Levine B. Methods in mammalian autophagy research. Cell. (2010) 140(3):313–26. doi: 10.1016/j.cell.2010.01.028 PMC2852113 20144757

[35] Bjørkøy G Lamark T Pankiv S Øvervatn A Brech A Johansen T. Chapter 12 monitoring autophagic degradation of p62/SQSTM1. Methods enzymology (2009) 452:181–97.e4. doi: 10.1016/S0076-6879(08)03612-4 19200883

[36] Gukovsky I Li N Todoric J Gukovskaya A Karin M Inflammation, Autophagy, and Obesity: Common features in the pathogenesis of pancreatitis and pancreatic cancer. Gastroenterology. (2013) 144(6):1199–1209.e4. doi: 10.1053/j.gastro.2013.02.007 PMC3786712 23622129

[37] Hsieh CW Chang CY Chen YM Chen HH Hung WT Gung NR. Impaired autophagic flux and its related inflammation in patients with adult-onset still’s disease. Oncotarget. (2018) 9(1):110–21. doi: 10.18632/oncotarget.23098 PMC5787422 29416600

[38] Spaulding CC Walford RL Effros RB. Calorie restriction inhibits the age-related dysregulation of the cytokines TNF-α and IL-6 in C3B10RF1 mice. Mech Ageing Dev (1997) 93(1-3):87–94. doi: 10.1016/S0047-6374(96)01824-6 9089573

[39] Rao SA Kunte AR. Interleukin-6: An early predictive marker for severity of acute pancreatitis. Indian J Crit Care Med (2017) 21(7):424–8. doi: 10.4103/ijccm.IJCCM_478_16 PMC5538089 28808361

[40] Mehta R Petrova A. Neutrophil function in neonates born to gestational diabetic mothers. J Perinatol (2005) 25(3):178–81. doi: 10.1038/sj.jp.7211241 15592426

[41] Qin B Zhou Z He J Yan C Ding S. IL-6 inhibits starvation-induced autophagy *via* the STAT3/Bcl-2 signaling pathway. Sci Rep (2015) 5(1):15701. doi: 10.1038/srep15701 26549519 PMC4637890

[42] Yao Z Zhang P Guo H Shi J Liu S Liu Y. RIP1 modulates death receptor mediated apoptosis and autophagy in macrophages. Mol Oncol (2015) 9(4):806–17. doi: 10.1016/j.molonc.2014.12.004 PMC5528779 25583602

[43] Cadwell K. Crosstalk between autophagy and inflammatory signalling pathways: balancing defence and homeostasis. Nat Rev Immunol (2016) 16(11):661–75. doi: 10.1038/nri.2016.100 PMC5343289 27694913

[44] Qian M Fang X Wang X. Autophagy and inflammation. Clin Trans Med (2017) 6:24. doi: 10.1186/s40169-017-0154-5 PMC5529308 28748360

[45] Canonico B Cesarini E Montanari M Di Sario G Campana R Galluzzi L. Rapamycin re-directs lysosome network, stimulates ER-remodeling, involving membrane CD317 and affecting exocytosis, in ampylobacter jejuni-lysate-infected U937 cells. Int J Mol Sci (2020) 21(6):2207. doi: 10.3390/ijms21062207 32210050 PMC7139683

[46] Shi Y Frankel A Radvanyi LG Penn LZ Miller RG Mills GB. Rapamycin enhances apoptosis and increases sensitivity to cisplatin *in vitro* . Cancer Res (1995) 55(9):1982–8. 7728769

[47] Yin J Gu L Wang Y Fan N Ma Y Peng Y. Rapamycin improves palmitate-induced ER stress/NF *κ* b pathways associated with stimulating autophagy in adipocytes. Mediators Inflamm (2015) 2015:1–12. doi: 10.1155/2015/272313 PMC4310475 25653476

[48] Wang JP Zhang MY. Role for target of rapamycin (mTOR) signal pathway in regulating neuronal injury after intracerebral hemorrhage. Cell Physiol Biochem (2017) 41(1):145–53. doi: 10.1159/000455983 28214828

[49] Carloni S Buonocore G Longini M Proietti F Balduini W. Inhibition of rapamycin-induced autophagy causes necrotic cell death associated with Bax/Bad mitochondrial translocation. Neuroscience. (2012) 203:160–9. doi: 10.1016/j.neuroscience.2011.12.021 22209856

[50] Fan YJ Zong WX. The cellular decision between apoptosis and autophagy. Chin J Cancer (2013) 32(3):121–9. doi: 10.5732/cjc.012.10106 PMC3845594 23114086

[51] Mei Q Zeng Y Huang C Zheng J Guo Y Fan J. Rapamycin alleviates hypertriglyceridemia-related acute pancreatitis *via* restoring autophagy flux and inhibiting endoplasmic reticulum stress. Inflammation. (2020) 43(4):1510–23. doi: 10.1007/s10753-020-01228-7 32642911

[52] Moulis M Vindis C. Methods for measuring autophagy in mice. Cells. (2017) 6(2):14. doi: 10.3390/cells6020014 28594368 PMC5492018

[53] Song S Tan J Miao Y Sun Z Zhang Q. Intermittent-hypoxia-induced autophagy activation through the ER-stress-related PERK/eIF2α/ATF4 pathway is a protective response to pancreatic β-cell apoptosis. Cell Physiol Biochem (2018) 51(6):2955–71. doi: 10.1159/000496047 30562747

[54] Windsor JA Hammodat H. Metabolic management of severe acute pancreatitis. World J Surg (2000) 24(6):664–72. doi: 10.1007/s002689910108 10773118

[55] Andriulli A Forlano R Napolitano G Conoscitore P. Pancreatic duct stents in the prophylaxis of post-ERCP pancreatic damage: A systematic analysis of benefits and associated risks. Digestion (2007) 75:156–63. doi: 10.1159/000106774 17684365

[56] van Brunschot S Bakker OJ Besselink MG Bollen TL Fockens P Gooszen HG. Treatment of necrotizing pancreatitis. Clin Gastroenterol Hepatol (2012) 10(11):1190–201. doi: 10.1016/j.cgh.2012.05.005 22610008

