# Supplementary figures and images for: Effect of calorie-restriction and rapamycin on autophagy and the severity of caerulein-induced experimental acute pancreatitis in mice
Source: Front Gastroenterol (Lausanne). 2022 Oct 12;1:977169. doi: 10.3389/fgstr.2022.977169 (PMC12952392; doi:10.3389/fgstr.2022.977169)

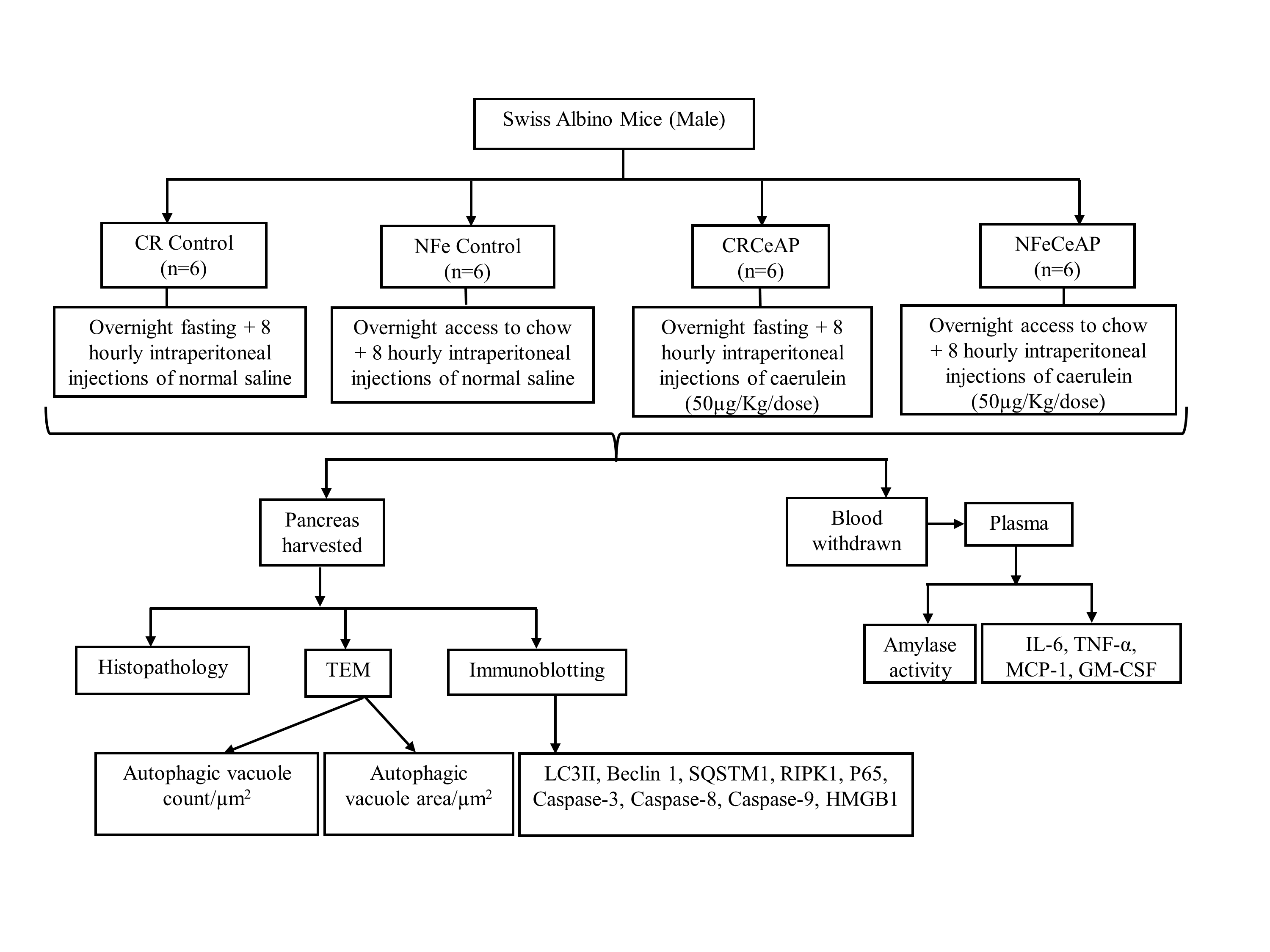

Supplement: Supplementary file 1 [file Image_1.tiff]

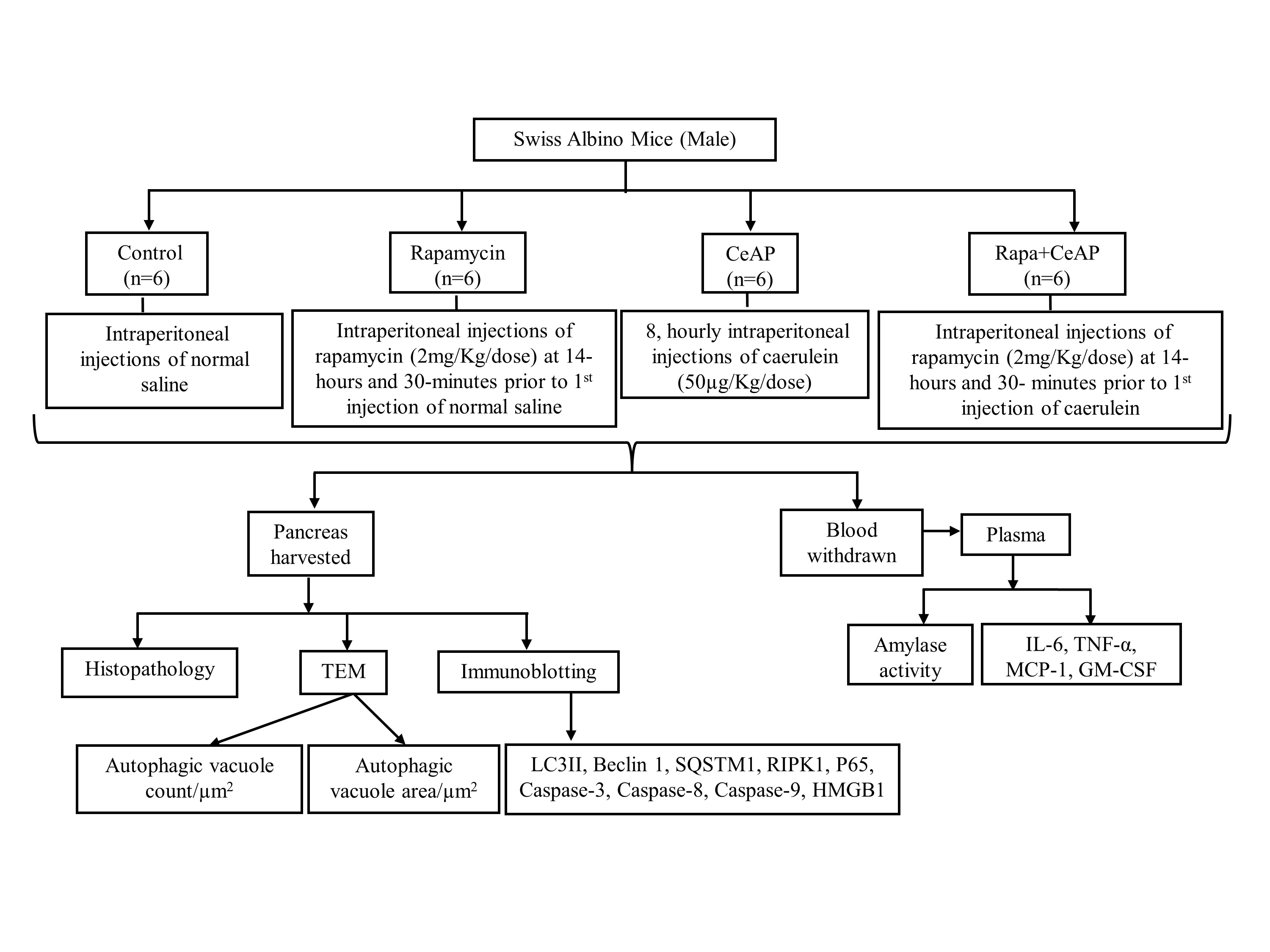

Supplement: Supplementary file 2 [file Image_2.tiff]
